# Regulation of AP-1 activity by the HTLV-2 APH-2 protein

**DOI:** 10.1186/1742-4690-8-S1-A161

**Published:** 2011-06-06

**Authors:** Céline Marban, William W Hall, Noreen Sheehy

**Affiliations:** 1 Centre for Research in Infectious Diseases, School of Medicine and Medical Science, University College Dublin, Dublin, Ireland

## 

In contrast to HTLV-1 which causes adult T-cell leukemia/lymphoma (ATL/ATLL) and HTLV-1 associated myelopathy/tropical spastic paraparesis (HAM/TSP), the role of HTLV-2 in human disease is less clearly defined but infection is associated with rare lympho-proliferative and neurological disorders. Transcription from the 3’ LTR of the HTLV-1 and HTLV-2 genomes governs the expression of two antisense regulatory proteins named HBZ (HTLV-1 basic leucine zipper) and APH-2 (antisense protein of HTLV-2), respectively. HBZ possesses a bZIP motif that facilitates its interaction with several cellular bZIP proteins including CREB2 and members of the AP-1 family such as c-Jun, JunB and JunD. These interactions inhibit Tax dependent LTR activation via CREB and AP-1 leading to the suggestion that HBZ counteracts the function of Tax 1 resulting in reduced viral gene expression and the enhancement of persistence in infected cells. Similarly, APH-2 inhibits Tax 2 mediated activation of the HTLV-2 LTR by interacting with CREB despite the absence of a bZIP motif. In the present study we investigated the transcriptional effects of APH-2 on the AP-1 pathway compared to HBZ. We clearly show that APH-2 stimulates basal AP-1-mediated transcription. In contrast to HBZ, we found that APH-2 enhances the ability of c-Jun to activate AP-1 activity. Co-immunoprecipitation assays demonstrate that APH-2 interacts with c-Jun in 293T cells. Furthermore as shown for HBZ, APH-2 down regulates Tax 2B mediated activation of AP-1 activity. These preliminary results suggest that HBZ and APH-2 may have distinct biological properties, which may contribute to the differential pathogenic potential of HTLV-1 and HTLV-2.

